# Gold nanoparticles as a potent radiosensitizer in neutron therapy

**DOI:** 10.18632/oncotarget.19837

**Published:** 2017-08-03

**Authors:** Eun Ho Kim, Mi-Sook Kim, Hyo Sook Song, Seung Hoon Yoo, Sei Sai, Kwangzoo Chung, Jiwon Sung, Youn Kyoung Jeong, YunHui Jo, Myonggeun Yoon

**Affiliations:** ^1^ Division of Heavy Ion Clinical Research, Korea Institute of Radiological and Medical Sciences, Seoul, Korea; ^2^ Department of Radiation Oncology, Korea Institute of Radiological and Medical Sciences, Seoul, Korea; ^3^ Department of Bio-convergence Engineering, Korea University, Seoul, Korea; ^4^ Department of Basic Medical Sciences for Radiation Damages, National Institute of Radiological Sciences, Chiba, Japan; ^5^ Department of Radiation Oncology, Samsung Medical Center, Seoul, Korea; ^6^ Research Center for Radiotherapy, Korea Institute of Radiological and Medical Sciences, Seoul, Korea

**Keywords:** gold nanoparticles, neutron therapy, radiosensitizer, cancer, γ-ray

## Abstract

The purpose of this study was to investigate the potential of gold nanoparticles as radiosensitizer for use in neutron therapy against hepatocellular carcinoma.

The hepatocellular carcinoma cells lines Huh7 and HepG2 were irradiated with γ and neutron radiation in the presence or absence of gold nanoparticles. Effects were evaluated by transmission electron microscopy, cell survival, cell cycle, DNA damage, migration, and invasiveness.

Gold nanoparticles significantly enhanced the radiosensitivity of Huh7 and HepG2 cells to γ-rays by 1.41- and 1.16-fold, respectively, and by 1.80- and 1.35-fold to neutron radiation, which has high linear energy transfer. Accordingly, exposure to neutron radiation in the presence of gold nanoparticles induced cell cycle arrest, DNA damage, and cell death to a significantly higher extent, and suppressed cell migration and invasiveness more robustly. These effects are presumably due to the ability of gold nanoparticles to amplify the effective dose from neutron radiation more efficiently.

The data suggest that gold nanoparticles may be clinically useful in combination therapy against hepatocellular carcinoma by enhancing the toxicity of radiation with high linear energy transfer.

## INTRODUCTION

Malignant tumors are a leading cause of mortality worldwide [1]. Almost 80% of patients with hepatocellular carcinoma (HCC) are from the Asia-Pacific region. HCCs are usually treated by surgical resection, with 5-year survival rates of 30–70% [2, 3]. However, surgery is suitable for fewer than 16% of patients, and conventional chemotherapy does not significantly improve clinical outcomes in patients with advanced tumors [4]. Radiotherapy may provide sustained local control in certain patients, an effect that may be enhanced by radiosensitizing agents [5]. In general, ionizing radiation kills cancer cells in two ways, depending on the energy of the radiation. Low-linear energy transfer (LET) radiation, such as X-rays, kills cells by generating reactive oxygen species (ROS) and free radicals [6], whereas high-LET radiation, such as neutrons, kills cells by nuclear interactions [7]. Because malignant tumors tend to have low oxygen levels, making them relatively unaffected by low-LET radiation [8], neutron radiation may be more appropriate. Indeed, neutron radiation has been shown to be more effective than low-LET radiation in treating salivary gland carcinomas, adenoid cystic carcinomas, and certain brain tumors, especially high-grade gliomas [7, 9]. In addition, neutron therapy generally requires shorter treatment cycles, as only one-third of the effective dose of neutrons is required to kill the same number of cancer cells as photons [7, 8].

Despite the promise of neutron therapy, it is still necessary to specifically increase toxicity to tumor cells while minimizing side effects in normal cells [10]. Recently, nanotechnology has provided both opportunities and challenges to improve cancer diagnosis and treatment [11], including the development of nanoscale radiosensitizers. In particular, gold nanoparticles, which passively accumulate in tumors [12–14], have shown promising results as radiosensitizers [15]. Although nanoparticles could result in damage to organelles and/or DNA, apoptosis, mutagenesis, and protein up/down regulation, the toxicity due to gold nanoparticles has been found to be minimal [16]. The advantages of gold nanoparticles include their high mass energy absorption due to a high atomic number (Z = 79) [17], their relatively easy synthesis, and their ready functionalization [18]. In general, biological molecules such as DNA and RNA are also capable of being functionalized by GNPs. This can be achieved by taking advantage of the electrostatic interactions between GNPs and their targeted biological molecule, thereby creating GNP bio-conjugates. Although gold nanoparticles were shown to act as radiosensitizers for X-rays, the effects of these particles on radiosensitivity have not been examined over a wide range of incident types of therapeutic radiation. This study therefore investigated the ability of gold nanoparticles to enhance the toxicity to neutron radiation of HCC cell lines *in vitro.* This study also attempted to determine the mechanisms driving the cellular response to high-LET uncharged radiation (neutrons) and low-LET radiation (photons).

## RESULTS

**Figure 1:**
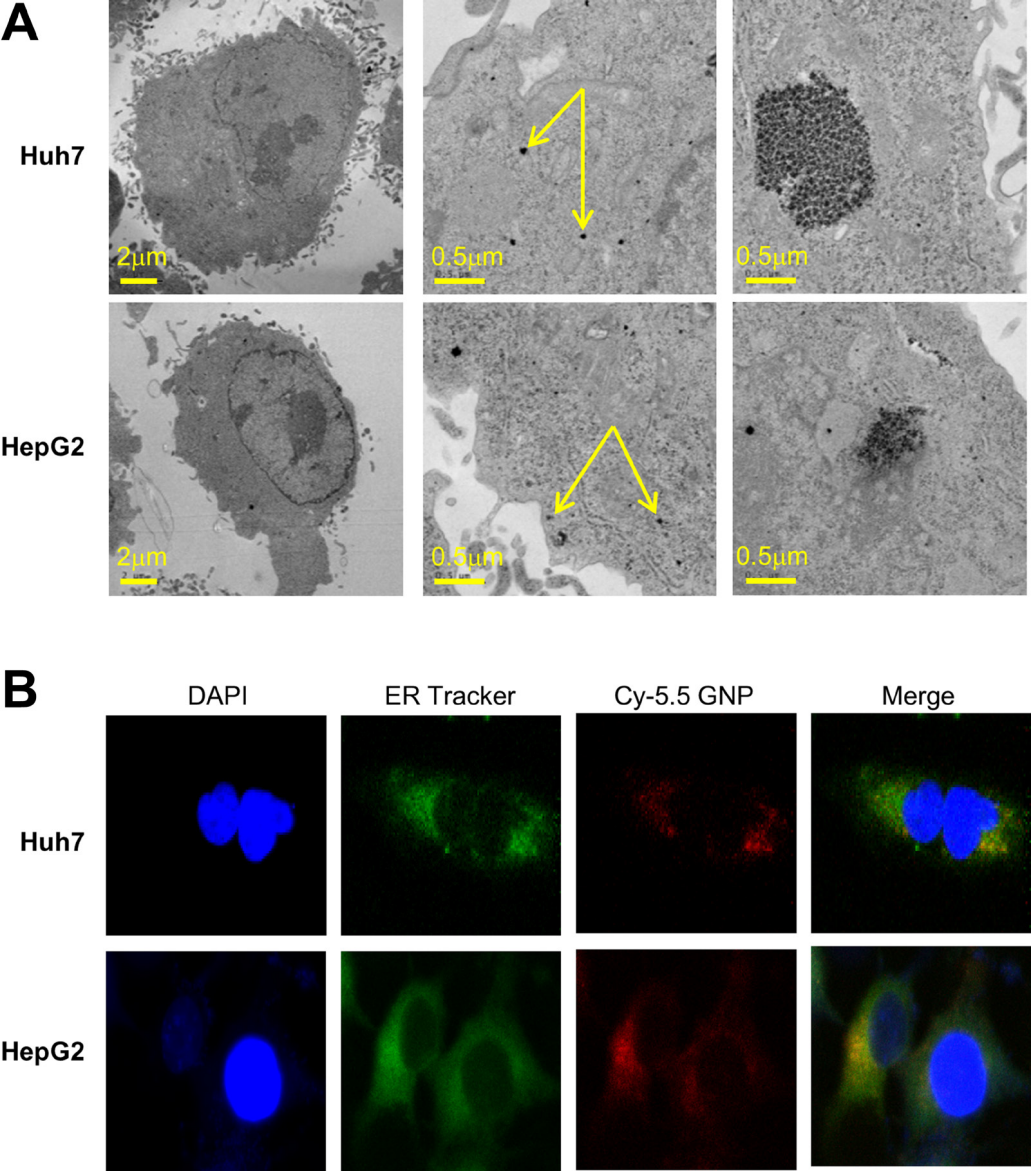
Intracellular localization of 5 nm gold particles (**A**) Transmission electron microscopy (80kV) of monodispersed 5 nm gold nanoparticles. (**B**) Huh7 and HepG2 cells incubated for 24 h with 1 mM Cy5.5-labeled gold nanoparticles, and stained with specific dyes for nuclei (DAPI) and endoplasmic reticulum.

**Figure 2:**
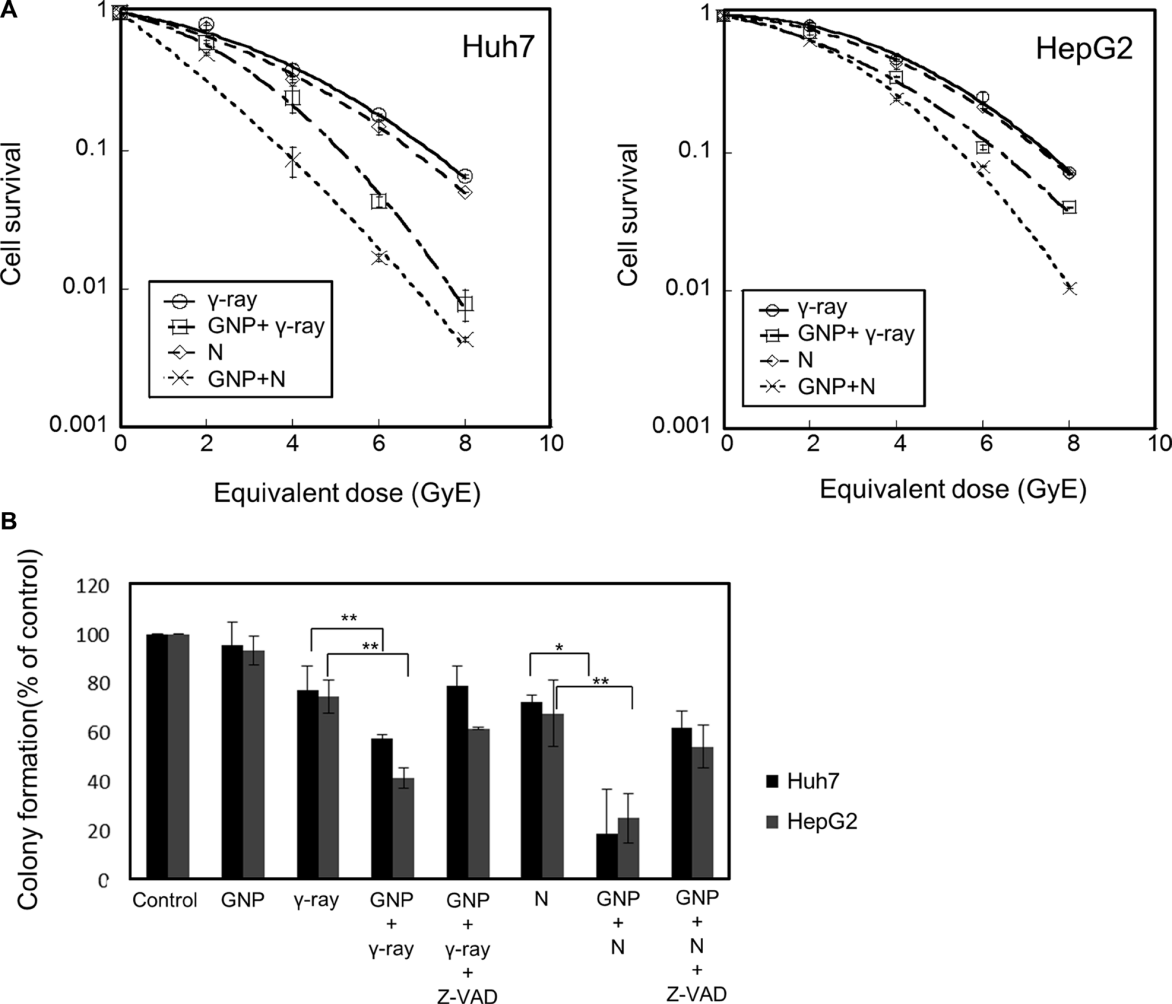

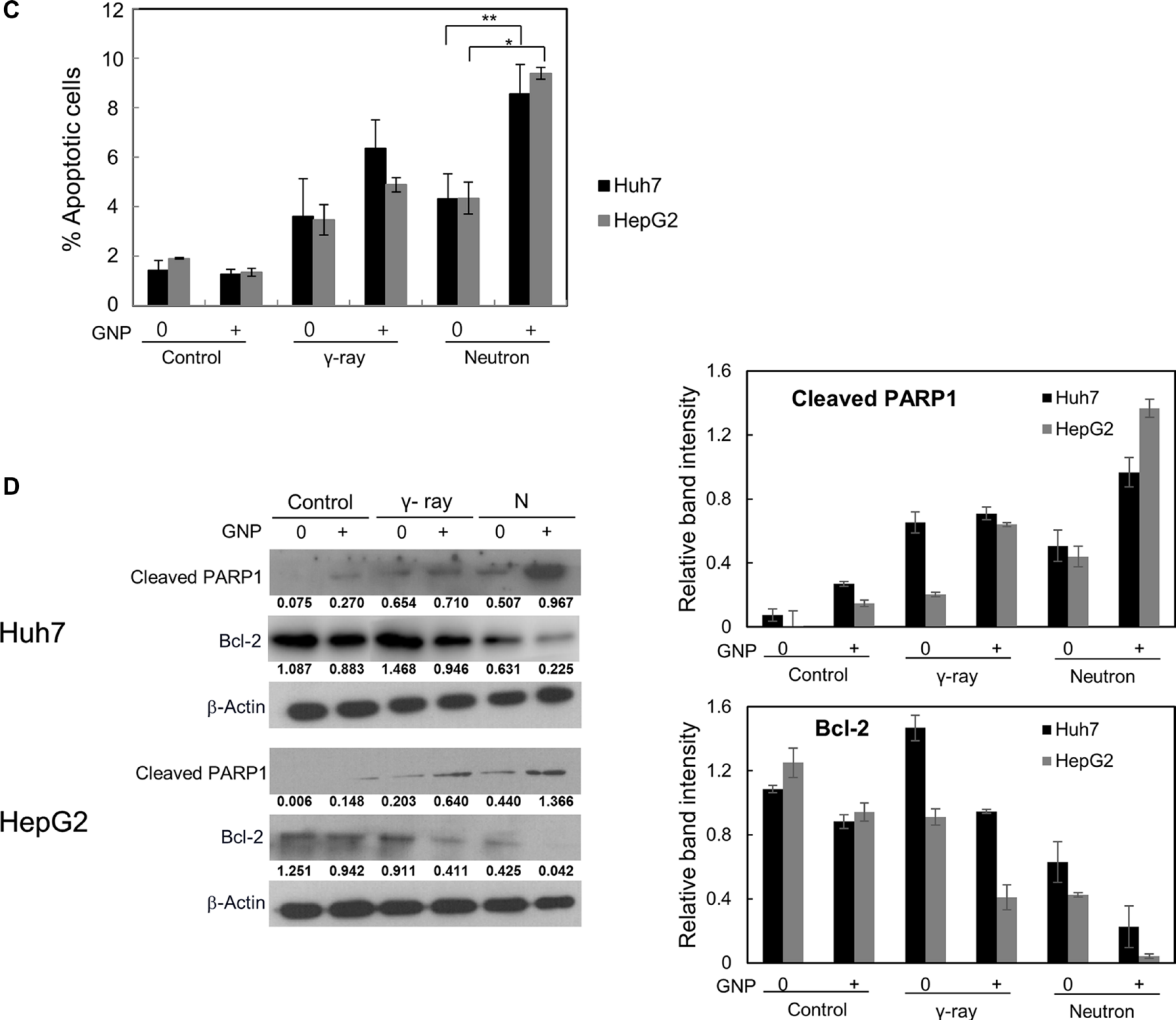
Radiosensitizing effects of gold nanoparticles (**A**) Colony-forming assays of Huh7 and HepG2 cells treated with 1 mM gold nanoparticles and irradiated with γ-rays and neutrons. Values are the means ± SD from three experiments. The x-axis shows the equivalent dose, expressed as GyE (Gray equivalent). (**B**) Partial abrogation of gold nanoparticle radiosensitization by a pan-caspase inhibitor. Cells were treated with 1 mM gold nanoparticles alone (4 h prior to radiation) or in combination with z-VAD-fmk (10 μM, 6 h prior to radiation). The absorbed doses were 5 Gy for γ-rays and 5 GyE for neutrons. Effect of z-VAD-fmk on gold nanoparticle radiosensitization was assessed by clonogenic survival assay. Values represent the means ± SDs of three experiments; ^*^P < 0.05, ^**^P < 0.001. (**C**) Apoptosis in Huh7 and HepG2 cells, as measured by annexin V staining 48 h after irradiation with 5 Gy of γ-rays or 5 GyE neutrons in the presence or absence of gold nanoparticles. Values represent the means ± SDs of three experiments; ^*^P < 0.05, ^**^P < 0.001. (**D**) Immunoblotting of cell lysates with indicated antibodies. The absorbed doses were 5 Gy for γ-rays and 5 GyE for neutrons. Band intensities for target proteins were normalized to that for β-actin. Values represent the means of 3 experiments ± SD.

Gold nanoparticles were taken up by HepG2 and Huh7 cells within 24 h (Figure 1A). Fluorescently labeled nanoparticles were similarly taken up, accumulating near the nuclear membrane and in the cytoplasm (Figure 1B). Co-staining indicated that most nanoparticles accumulated in the endoplasmic reticulum (Figure 1B). Cells irradiated in the presence of gold nanoparticles had a significantly lower survival rate than cells irradiated in the absence of nanoparticles (Figure 2A). The parameters of the linear quadratic fitting of survival curves and the doses required to reduce survival to 10% are shown in the tables (Tables 1, 2).

**Table 1:** Linear quadratic fitting parameters α and β for survival curves in cells irradiated in the presence or absence of gold nanoparticles (GNP) and incubated for 14 days

Cell type	Treatment	α (Gy^-1^)	β (Gy^-1^)
Huh7	γ-ray	0.106 ± 0.379	0.029 ± 0.055
	γ-ray + GNP	0.154 ± 0.399	0.057 ± 0.059
	Neutron	0.138 ± 0.378	0.029 ± 0.055
	Neutron + GNP	0.525 ± 0.413	0.021 ± 0.061
HepG2	γ-ray	0.004 ± 0.375	0.040 ± 0.053
	γ-ray + GNP	0.134 ± 0.401	0.034 ± 0.059
	Neutron	0.043 ± 0.375	0.036 ± 0.055
	Neutron + GNP	0.093 ± 0.367	0.059 ± 0.053

**Table 2:** Radiation dose needed to kill 90% of cells (D10) in the presence or absence of gold nanoparticles (GNP)

Cell type	Radiation	D10 without GNP	D10 with GNP
Huh7	γ-ray	7.26 Gy	5.14 Gy
	Neutron	6.84 Gy	3.81 Gy
HepG2	γ-ray	7.53 Gy	6.49 Gy
	Neutron	7.42 Gy	5.50 Gy

Values were obtained from Figure 2A.

To test if caspase activation, which leads to apoptosis induction, is the main cause of GNP-induced radiosensitization, Huh7 and HepG2 cells were incubated in the presence or absence of the apoptosis inhibitor z-VAD-fmk, which inactivates caspases, and the results of clonogenic assays were analyzed. Treatment with z-VAD-fmk significantly blocked the increased apoptosis of these cells induced by GNP plus radiation (Figure 2B). Irradiation of the two HCC cell lines in the presence of gold nanoparticles significantly increased the numbers of apoptotic cells (Figure 2C and Table 3). Compared with radiation alone, combined treatment enhanced PARP1 fragmentation and reduced the expression of the anti-apoptotic protein Bcl-2 (Figure 2D), confirming that gold nanoparticles enhanced apoptosis. In addition, the effect of gold nanoparticles was more pronounced with neutrons than with γ radiation.

**Table 3:** Detection of apoptotic cells by annexin V staining on 2 HCC cells

Cell type	Treatment	% Apoptotic cells	Ratio
Huh7	Control GNP γ-ray γ-ray+GNP Neutron Neutron+GNP γ-ray+GNP/GNP γ-ray+GNP/γ-ray Neutron+GNP/GNP Neutron+GNP/Neutron	1.43 1.28 3.62 6.36 4.33 8.57	4.97 1.76 6.70 1.98
HepG2	Control GNP γ-ray γ-ray+GNP Neutron Neutron+GNP γ-ray+GNP/GNP γ-ray+GNP/γ-ray Neutron+GNP/GNP Neutron+GNP/Neutron	1.91 1.36 3.47 4.89 4.35 9.40	3.60 1.41 6.91 2.16

**Figure 3:**
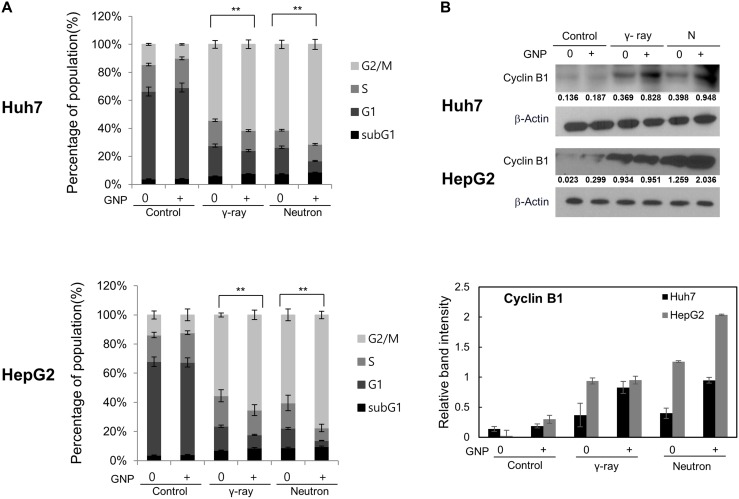
Irradiation in the presence of gold nanoparticles modulates cell cycle progression and expression of cell cycle regulators (**A**) Cell cycle distribution, irradiated with 5 Gy of γ and 5 GyE of neutron radiation and (**B**) expression of cyclin B1 in Huh7 and HepG2 cells treated with 1 mM gold nanoparticles, irradiated with 5 Gy of γ and 5 GyE of neutron radiation, and incubated for 24 h. Band intensities for target proteins were normalized to that for β-actin. Values represent the means of 3 experiments ± SD.

GNP treatment itself did not alter cell cycle distribution at 24 h (Figure 3). Conversely, γ-ray or neutron radiation alone markedly increased the number of cells in G2/M and reduced the cells in G1 (Figure 3A) [19–21], while also reducing the number of cells in S-phase, albeit to a lesser extent than the reduction in G1 phase. Combination treatment of both cell lines caused the greatest accumulation of cells in G2/M phase, suggesting efficient induction of cell cycle arrest in both. Similarly, western blotting showed that radiation alone or combined treatment induced significant accumulation of cyclin B, a key regulator of G2/M transition (Figure 3B). Although not statistically significant, the ability of GNPs to alter cell cycle distribution was more pronounced with neutrons than with γ radiation (Figure 3).

**Figure 4:**
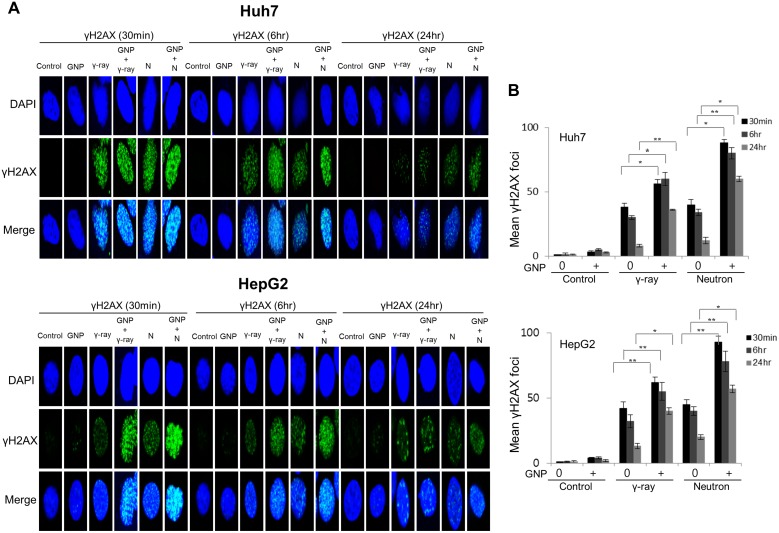

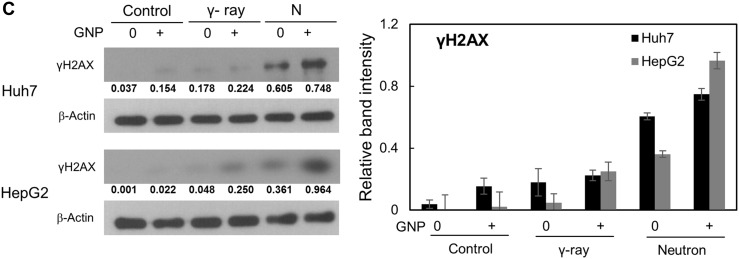
Gold nanoparticles enhance radiation-induced DNA damage (**A**, **B**) Immunocytochemistry staining for phosphorylated H2AX, a marker of DNA damage response, in Huh7 and HepG2 cells exposed to γ-rays (5 Gy) and neutron radiation (5 GyE) in the absence or presence of gold nanoparticles, assessed 30 min, 6 h and 24 h after irradiation. (**C**) Immunoblotting of cell lysates with indicated antibodies. The absorbed doses were 5 Gy for γ-rays and 5 GyE for neutrons. Band intensities for target proteins were normalized to that for β-actin. Values represent the means of 3 experiments ± SD.

Damage to DNA foci occurred within 30 min and 6 h after treatment of the HCC cell lines with and without GNPs, respectively, with gamma radiation (5 Gy) or neutron radiation (5 GyE), with this damage persisting for up to 24 h. More foci were observed after neutron than after γ-radiation of GNP-treated cells (Figure 4A, 4B and Supplementary Figure 1A, 1B. In addition, sustained expression of phosphorylated H2AX, a marker of DNA damage response, by cells treated with irradiation plus GNPs was observed 24 h later, both by immunofluorescence (Figure 4A, 4B) and western blotting (Figure 4C).

**Figure 5:**
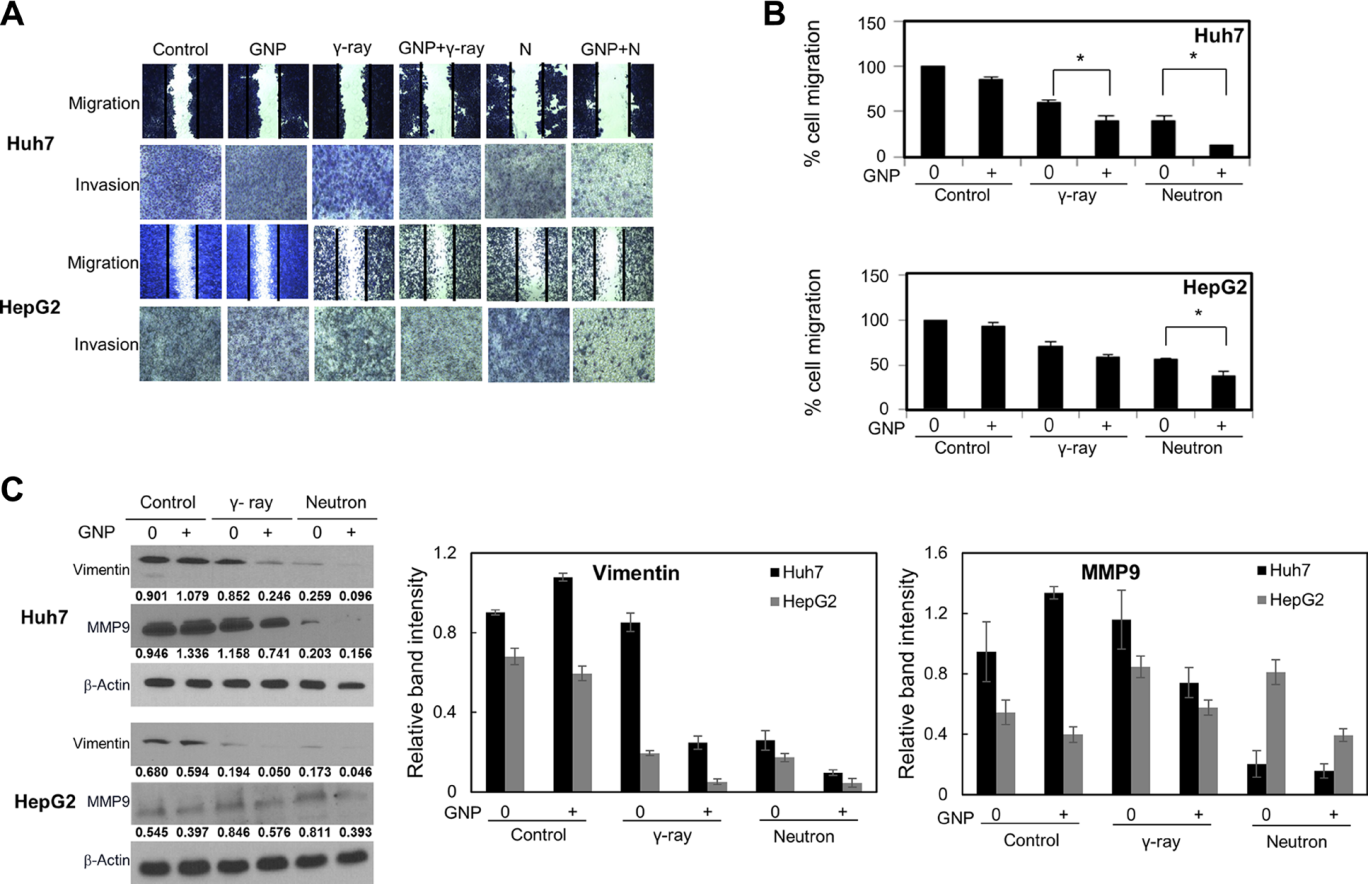

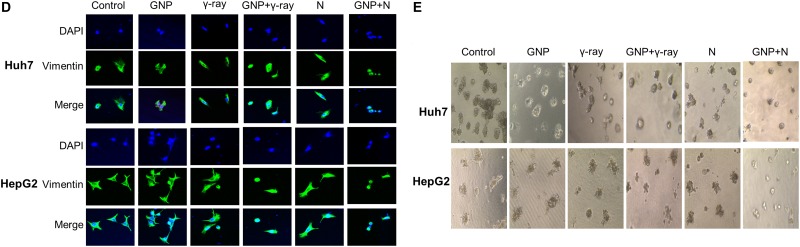
Effect of gold nanoparticles and radiation on cell migration and invasiveness (**A**, **B**) Plates from the scratch assay were photographed, distances between migrating cell fronts were measured, and the fraction of cells that had migrated was calculated (upper). Values are mean ± SD of three experiments. Cells exposed to γ-rays and neutron radiation (5 Gy, 5 GyE). Cell invasion was examined by Matrigel transwell chamber assay (lower). (**C**) Immunoblotting of cell lysates with indicated antibodies. Cells exposed to γ-rays and neutron radiation (5 Gy, 5 GyE). Band intensities for target proteins were normalized to that for β-actin. Values represent the means of 3 experiments ± SD. (**D**) Immunocytochemistry staining for Vimentin in Huh7 and HepG2 cells exposed to γ-rays and neutron radiation (5 Gy, 5 GyE) in the absence or presence of gold nanoparticles. (**E**) 3D spheroid growth assay of Huh7 and HepG2 cells treated with gold nanoparticles and radiation for four days. Phase-contrast images indicated that untreated cells formed polarized spheroids, but cells exposed to gold nanoparticles and radiation did not. Cells exposed to γ-rays and neutron radiation (5 Gy, 5 GyE).

The combination of GNPs and radiation significantly inhibited cell migration and invasion (Figure 5A, 5B), as well as suppressing the expression of proteins, including vimentin and MMP-9, that drive epithelial-mesenchymal transition and invasion (Figure 5C). In particular, vimentin expression was markedly reduced in cells treated with GNPs and neutron radiation (Figure 5D). GNP treatment itself did not alter cell migration or invasion. In addition, irradiation in the presence of GNPs reduced stellate structures that are a hallmark of invasive cells (Figure 5E). These effects were more pronounced with neutron radiation than with γ-rays.

## DISCUSSION

GNPs are promising nanoscale drug carriers, radiosensitizers, and imaging contrast enhancers for cancer diagnosis and therapy [11, 15, 22–24]. These applications are based on size-dependent passive targeting, and on the physical and chemical properties of gold [11, 12, 22, 25], which include dose enhancement, as seen in Monte Carlo simulations [6, 26–29]. Indeed, the radiobiological effects of GNPs have been extensively investigated for various types of therapeutic radiation to determine their potential clinical applications [30–33]. Based on the comparison study using protons in combination with GNPs versus using protons alone, Kim et al. [32] and Polf et al. [33] have shown an over 50% increase of one-year survival in mice and an approximately 15% increase in cell killing of prostate cancer cell lines. Recently, Kaur et al. [31] have shown that the dose of carbon ion needed for 90% cell killing in GNP treated HeLa cells was 2.3 Gy which shows approximately 28% reduction of dose for GNP treated cells as compared to control cells. To date, however, these studies have been restricted to low-LET radiation or charged particle irradiation. The present study therefore characterized the potential of GNPs as radiosensitizers for high-LET uncharged particle, i.e., neutron, irradiation.

Our results suggested that GNPs enhance the radiotoxicity of neutron radiation more significantly than γ-ray radiation, as measured by apoptosis, cell cycle arrest, DNA damage, and metastatic potential. To evaluate the radiosensitizing effects of GNPs, the radiosensitivity enhancement ratio (REF) was calculated as the dose (Gy) of radiation alone divided by the dose of radiation plus GNPs that resulted in 10% cell survival (D10) (Table 4). While REF values resulting from the addition of GNPs to Huh7 and HepG2 cells were 1.41 and 1.16, respectively, for gamma irradiation, they were 1.80 and 1.35, respectively, for neutron irradiation. Our results also suggested that GNP alone did not yield foci, even 24 h after exposure, suggesting that GNP treatment itself did not alter the induction or subsequent disappearance of foci at any time point examined. The combination of GNPs with γ-ray and neutron irradiation caused much greater DNA damage to HCC cells than γ-ray and neutron irradiation alone (Table 5), with the REF being higher for neutrons than for γ-rays.

**Table 4:** Radiosensitivity enhancement factor (REF) and dose reduction

Cell type	Radiation	REF	Dose reduction (%)
Huh7	γ-ray	1.41	29.2
	Neutron	1.80	44.3
HepG2	γ-ray	1.16	13.8
	Neutron	1.35	25.9

Values were obtained from Figure 2A.

REF: ratio of the radiation dose required to kill 90% of cells in the presence of gold nanoparticles divided by the dose in the absence of gold nanoparticles.

**Table 5:** Detection of γH2AX foci on 2 HCC cells

Cell type	Treatment time	Treatment	γH2AX foci number
Huh7	30 min	γ-ray+GNP /γ-ray /GNP Neutron+GNP/ Neutron /GNP	56/38/3.5 88/40/3.5
	6 hr	γ-ray+GNP /γ-ray /GNP Neutron+GNP/ Neutron /GNP	60/30/5.0 80/34/5.0
	24 hr	γ-ray+GNP /γ-ray /GNP Neutron+GNP/ Neutron /GNP	36/8.0/3.0 60/12/3.0
HepG2	30 min	γ-ray+GNP /γ-ray /GNP Neutron+GNP/ Neutron /GNP	62/42/4.0 93/45/4.0
	6 hr	γ-ray+GNP /γ-ray /GNP Neutron+GNP/ Neutron /GNP	55/32/4.0 78/40/4.0
	24 hr	γ-ray+GNP /γ-ray /GNP Neutron+GNP/ Neutron /GNP	40/13/2.0 57/20/2.0

Low energy photon irradiation of a material containing GNPs has been reported to enhance the radiosensitivity of the material by producing secondary electrons from the nanoparticles due to the high atomic number of gold [6]. These secondary electrons are generated from GNPs by photoelectric effects and Auger cascades, with the latter considered the major source of dose enhancement. Because of their low kinetic energy and low speed, Auger electrons seem to transfer all of their kinetic energy over a short range, locally generating high concentrations of hydroxyl radicals (.OH) and thereby amplifying the effective dose [34, 35]. Although the exact mechanism underlying the radiosensitizing effect of GNPs has not been firmly established, the biological effects of neutrons can be explained by the interaction of recoil protons ([36] and references therein). Most (60%) of the neutrons used in this experiment were fast neutrons (1–20 MeV), with the percentage rising to 80% percent if relativistic neutrons (> 20 MeV) are included. As for the neutron cross section with GNPs in this energy range, hadronic elastic scattering is dominant and, especially for relativistic neutrons, 3-neutron generation cross sections are significantly enhanced [37]. Therefore, by interacting with GNPs, more neutrons will be generated in reaction chains, with the increase in neutron population likely contributing to their biological effects. (We are currently expanding this mechanistic investigation by performing a Monte Carlos simulation.)

Although further research is needed to determine whether nanoparticles enhance radiosensitivity to neutron therapy, its applications are highly limited. For example, only three centers in the world currently treat cancer with fast neutrons, perhaps because of a lack of funding and issues of regulatory approval. An alternative approach may be to use other forms of high-LET radiation, such as carbon beams, which are used in Japan and Europe. Investigations are therefore needed to assess whether nanoparticles alter radiosensitivity to carbon beams, and to evaluate the safety of such approaches. Finally, although our results suggest that GNPs have potential as radiosensitizers in neutron therapy, *in vivo* experiments in animal models are necessary to minimize possible clinical complications.

## MATERIALS AND METHODS

### Sample preparation

Huh7 and HepG2 HCC cells were cultured in RPMI 1640 medium (WelGene, Daegu, Korea), supplemented with 10% (v/v) fetal bovine serum (Lonza, MD, USA) and 1% (v/v) penicillin-streptomycin (Lonza, MD, USA) at 37°C in a humidified incubator containing 5% CO_2_. Antibodies against Bcl2, cyclin B, vimentin, and β–actin were purchased from Santa Cruz Biotechnology (Santa Cruz, CA, USA). Antibodies against cleaved poly(ADP-ribose) polymerase-1 (PARP1) and MMP9 were obtained from Cell Signaling Technology (Danvers, MA, USA), and antibodies against phosphorylated H2AX from Millipore (Billerica, MA, USA). Gold nanoparticles, about 5 nm in diameter, were purchased from Cytodiagnostics (Burlington, ON, Canada).

### Irradiation and visualization of gold nanoparticles

HCC cells were seeded on glass cover slips and incubated at 37°C for 24 h in fresh medium with or without gold nanoparticles. Fluorescently labeled ER tracker (Invitrogen, CA, USA), Cy5.5 (Nanocs, Eugene, OR, USA) and DAPI (Thermo Fisher Scientific, MA, USA) were added and colocalization analysis was performed using confocal microscopy [17]. To determine the intracellular distribution of gold nanoparticles, cells were seeded in a culture dish, allowed to adhere for one day, incubated for another 24 h at 37°C with 1 mM gold nanoparticles, and imaged by transmission electron microscopy as described [11].

Cells with/without gold nanoparticles were irradiated with a ^137^Cs γ-ray source (Atomic Energy of Canada, Ltd., Ontario, Canada), at a dose of 3.81 Gy/min, or with fast neutrons (Average energy: 9.8 MeV, Approximate LET: 30-40 keV/μm) which were produced by bombarding beryllium with ^9^Be(p,n)^10^B protons in an MC-50 cyclotron (Scanditronix, Uppsala, Sweden). Gray equivalent (GyE) unit for neutron irradiation states the equivalent biological dose compared to x-ray therapy and was found experimentally using in-vitro study. In all experiments involving gold nanoparticle treated cells, the nanoparticles were added before irradiation and were mixed with culture medium until the end of biological analysis.

### Biological analysis

### Colony-forming assays, flow cytometry and apoptosis analysis

Cells mixed with 1 mM gold nanoparticles were irradiated and incubated for 14 days, as described, and the resulting colonies were stained with 0.4% crystal violet (Sigma, St. Louis, MO, USA) [38]. Cells were cultured, harvested at indicated time points, stained with 1 μg/mL propidium iodide (Sigma, MO, USA), and sorted using a FACScan flow cytometer, with data analyzed by CellQuest (both from Becton Dickinson, CA, USA). Cell apoptosis was assayed as described [12].

### Immunocytochemistry and western blotting

Cells were grown in chambered slides, allowed to attach for one day, irradiated in the presence or absence of gold nanoparticles, and analyzed essentially as described [17]. For western blotting experiments, pretreated cells with/without gold nanoparticles were irradiated with gamma rays or neutrons, incubated for 24 h, lysed with RIPA buffer, and immunoblotted as described [15].

### Cell migration, transwell chamber invasion and 3D spheroid growth assays

To assess cell migration, cells were grown to ~90% confluency in 6-well plates, and the layer of cells was finely scratched with a sterile pipette tip. Invasion was measured *in vitro* using transwell chambers, according to the manufacturer's protocol. Cells that passed through Matrigel-coated membranes were stained with a crystal violet solution supplied in the transwell invasion assay kit (Chemicon, Millipore, MA, USA) and photographed after 24 h. For 3D-spheroid growth assays, cells suspended in 2.5% Matrigel were added to 48-well plates coated with Matrigel, and allowed to grow for up to 4 days.

### Statistical analysis

Statistical significance was determined by Student's *t*-test. Differences were considered significant at *p* values less than 0.05 and 0.001.

## SUPPLEMENTARY MATERIALS AND FIGURES


