# Supplementary material for: Gold nanoparticles as a potent radiosensitizer in neutron therapy
Source: Oncotarget. 2017 Aug 3;8(68):112390–400. doi: 10.18632/oncotarget.19837 (PMC5762518; doi:10.18632/oncotarget.19837)
Supplement: Supplementary file 1 [file oncotarget-08-112390-s001.pdf]

Gold nanoparticles as a potent radiosensitizer in neutron therapy

SUPPLEMENTARY MATERIALS

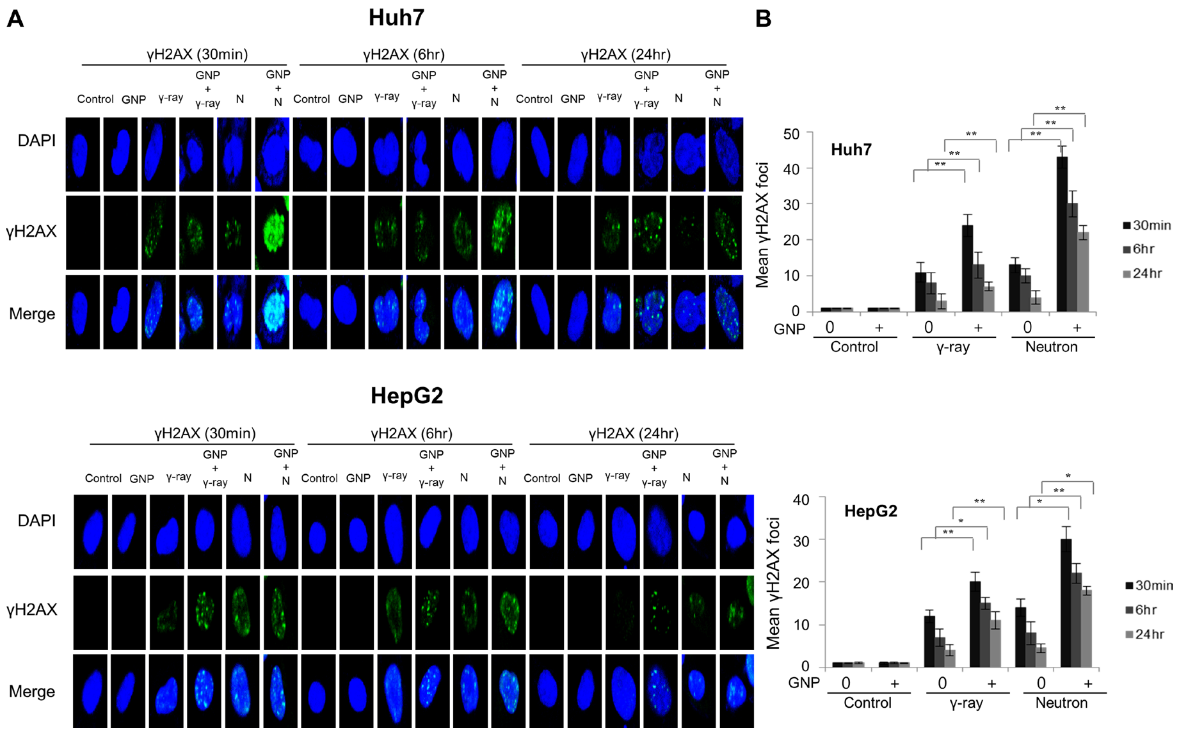

Supplementary Figure 1: Gold nanoparticles enhance low dose radiation-induced DNA damage. (**A**, **B**) Immunocytochemistry staining for phosphorylated H2AX, a marker of DNA damage response, in Huh7 and HepG2 cells exposed to γ-rays and neutron radiation (1 Gy, 1 GyE) in the absence or presence of gold nanoparticles, and results 30 min, 6 h and 24 h after irradiation.
